# Eignung rettungsdienstlicher Sichtungskategorien zur Vorhersage des ambulanten oder stationären Weiterversorgungsbedarfs: eine explorative Analyse von Routinedaten

**DOI:** 10.1007/s00063-025-01300-w

**Published:** 2025-06-25

**Authors:** Torben Brod, Uta Hillebrand, Christoph Schröder, Andreas Flemming, Nils Schneider, Tanja Schleef

**Affiliations:** 1 Zentrale Notaufnahme, Medizinische Hochschule Hannover, Carl-Neuberg-Straße 1, 30625 Hannover, Deutschland; 2 Klinik für Nieren und Hochdruckerkrankungen, Medizinische Hochschule Hannover, Hannover, Deutschland; 3 Stabsstelle für Interdisziplinäre Notfall- und Katastrophenmedizin (INKM), Medizinische Hochschule Hannover, Hannover, Deutschland; 4 Institut für Allgemeinmedizin und Palliativmedizin, Medizinische Hochschule Hannover, Hannover, Deutschland

**Keywords:** Notfallversorgung, Rettungsdienst, Ambulante Behandlung, Dringlichkeitseinschätzung, Versorgungsforschung, Emergency care, Prehospital emergency medicine, Outpatient care, Triage, Health services research

## Abstract

**Hintergrund:**

Eine effiziente und patientenzentrierte Notfallversorgung erfordert eine frühzeitige Lenkung des Patientenstroms. Ob dies bereits präklinisch durch den Rettungsdienst möglich ist und zu einer Entlastung der Notaufnahmen beitragen kann, ist unklar.

**Ziel der Arbeit:**

Untersuchung der Übereinstimmung von rettungsdienstlicher Sichtungskategorie und tatsächlicher Art der Weiterversorgung (ambulant/stationär); bei ambulant verbliebenen Patienten weiterhin Evaluation des innerklinischen Ressourcenverbrauchs.

**Methoden:**

Analyse präklinischer und klinischer Routinedaten aller Patienten, die innerhalb von 2 Wochen per Rettungsdienst in die Notaufnahme einer Universitätsklinik eingeliefert wurden. Die Analysen erfolgten deskriptiv und mittels Mann-Whitney-U-Test bzw. χ^2^-Test. Zusätzlich wurden positive und negative prädiktive Werte ermittelt.

**Ergebnisse:**

Im Beobachtungszeitraum wurden 570 Patienten per Rettungsdienst eingeliefert, von denen 307 (53,9 %) ambulant verblieben. Für 309 Patienten (54,2 %) stimmte die rettungsdienstliche Sichtungskategorie mit der Art der Weiterversorgung (ambulant/stationär) überein, in 249 Fällen (43,7 %) wurde der stationäre Behandlungsbedarf überschätzt. 262 Patienten (85,3 %) erhielten vor Entlassung mindestens eine diagnostische bzw. therapeutische Ressource, am häufigsten Röntgenaufnahmen (118/38,4 %).

**Diskussion:**

Nur in etwa der Hälfte der Fälle stimmte die rettungsdienstliche Prognose mit der tatsächlichen Art der Weiterversorgung überein. Auch ambulant verbliebene Rettungsdienstpatienten erhielten zumeist weiterführende diagnostische und therapeutische Ressourcen. Eine direkte Zuweisung rettungsdienstlicher Patienten in alternative Versorgungsstrukturen erscheint auf Basis ihres Ressourcenbedarfs daher risikobehaftet.

## Abstract (en)

**Background:**

Efficient and patient-centered emergency care requires early control of patient flow. It is unclear whether this is already possible in the prehospital phase by emergency medical services (EMS) and whether this can contribute to relieving pressure on emergency departments (EDs).

**Objectives:**

To examine the correlation between prehospital triage categories by EMS personnel and patient admission/discharge after treatment in the ED, and to assess the use of hospital resources for patients who remained outpatients.

**Methods:**

Retrospective analysis of routine preclinical and clinical data from all patients presenting to the ED of a university hospital by EMS over a 2-week period. Analyses were performed descriptively and using the Mann–Whitney U test and the χ^2^ test. Positive and negative predictive values were also calculated.

**Results:**

During the observation period, 570 EMS patients were seen in the ED, of whom 307 (53.9%) remained as outpatients. For 309 (54.2%) of the patients, the triage category assigned by the EMS corresponded to the type of subsequent care (outpatient/inpatient); in 249 cases (43.7%) the need for inpatient care was overestimated. In addition, 262 patients (85.3%) received at least one diagnostic or therapeutic resource prior to discharge, the most common being X‑rays (118/38.4%).

**Conclusion:**

In only about half of the cases was the EMS personnel’s prognosis consistent with the subsequent decision to admit or discharge the patient from the ED. Patients who remained as outpatients tended to require further diagnostic and therapeutic resources. Therefore, direct transfer of EMS patients to alternative care structures seems risky in the current environment.

Um eine bedarfsgerechte Notfallversorgung bei effizienter Ressourcennutzung zu gewährleisten, ist eine frühzeitige Lenkung des Patientenstroms bereits durch den Rettungsdienst essenziell. Die Zuweisung in ein Krankenhaus sollte hierbei sowohl den individuellen Versorgungsbedarf der Patienten als auch die Kapazitäten des Zielkrankenhauses berücksichtigen, die durch die Notfallversorgungsstufe, die aktuelle Auslastung der Notaufnahme und die stationären Bettenkapazitäten begrenzt sein können.

Die Notfallversorgung in Deutschland ist in 3 jeweils eigenständige Bereiche gegliedert, von denen der Rettungsdienst eine entscheidende Rolle bei der Erstversorgung und Zuführung von Patienten in die Notaufnahmen der Krankenhäuser übernimmt [24]. Um eine zielgerichtete Krankenhauszuweisung zu gewährleisten, wurden u. a. elektronische Dispositionshilfen (z. B. der webbasierte Interdisziplinäre Versorgungsnachweis IVENA eHealth (mainis IT-Service GmbH, Offenbach am Rhein), Rescuetrack (rescuetrack GmbH, Reutlingen) oder MediRIG (Landesbetrieb Information und Technik Nordrhein-Westfalen IT.NRW)) entwickelt und deren verpflichtende Nutzung bereits in einigen Bundesländern im Landeskrankenhausgesetz verankert (z. B. Nds. Krankenhausgesetz). Mittels dieser können sich die Mitarbeiter des Rettungsdiensts in Echtzeit über die aktuellen Behandlungs- und Versorgungsmöglichkeiten von Notaufnahmen und Krankenhäusern informieren [3, 10, 20]. Für eine effiziente und ressourcenschonende Nutzung der Versorgungskapazitäten sowie zur Gewährleistung der Patientensicherheit ist bereits am Einsatzort eine möglichst präzise Ersteinschätzung der Patienten erforderlich, bei der die Zuweisung einer geeigneten Sichtungskategorie erfolgt. Die systemdefinierten Sichtungskategorien sollen bereits präklinisch eine Einschätzung über die Behandlungsdringlichkeit sowie den stationären bzw. ambulanten Verbleib der Patienten geben. Innerhalb des IVENA-Systems werden 3 Sichtungskategorien (SK) unterschieden [10, 22]:

- SK1: Notfall, sofortige Intervention im Krankenhaus/sofortiger Arztkontakt erforderlich;
- SK2: dringende Behandlung, stationäre Aufnahme wahrscheinlich erforderlich, aber keine unmittelbare Intervention notwendig;
- SK3: nichtdringende Behandlung, stationäre Aufnahme wahrscheinlich nicht erforderlich, aber Versorgung im Krankenhaus notwendig.

Angesichts der angespannten Situation in den Notaufnahmen mit regelhaften Überlastungen von strukturellen wie personellen Kapazitäten [23] werden frühzeitige und korrekte Allokationsentscheidungen sowie präferierte Versorgungspfade zur Abklärung und Behandlung gefordert [21]. Erste Studien haben hierbei das Potenzial zur Umsteuerung niedrigdringlicher Rettungsdienstpatienten in die ambulante Versorgung untersucht, ohne jedoch die erforderlichen diagnostischen und therapeutischen Ressourcen für eine abschließende Entscheidungsfindung zu berücksichtigen [9, 15].

Ziel dieser Arbeit ist es, die Vorhersagbarkeit des Patientenverbleibs (ambulant/stationär) anhand der Übereinstimmung zwischen rettungsdienstlicher Sichtungskategorie und tatsächlicher Weiterversorgung zu analysieren. Weiterhin soll anhand des innerklinischen Ressourcenverbrauchs in der Notaufnahme untersucht werden, ob eine bereits präklinische Disposition der ambulant verbliebenden Patienten in die vertragsärztliche Versorgung möglich gewesen wäre.

## Methoden

### Studiendesign

Es wurde eine retrospektive Analyse aller vom 01.10.2022 bis zum 14.10.2022 per Rettungsdienst in die Zentrale Notaufnahme (ZNA) der Medizinischen Hochschule Hannover zugewiesenen Patienten durchgeführt. Ausgeschlossen wurden Kinder und Jugendliche mit nichttraumatischen Vorstellungsgründen, Patientinnen der Geburtshilfe sowie Patienten der Zahnklinik, da diese über eigenständige Notfallversorgungsbereiche verfügen. Die Fallzahl ergab sich aus der Anzahl der rettungsdienstlich zugewiesenen Patienten. Da es sich um eine rein explorative Studie handelte, wurde auf eine formale Fallzahlberechnung verzichtet. Ein positives Votum der Ethikkommission (Nr. 10937_BO_K_2023) sowie eine lokale datenschutzrechtliche Genehmigung (05.07.2023) lag vor.

### Datenbasis

Es erfolgte eine Extraktion von präklinischen Routinedaten aus dem IVENA-System zur rettungsdienstlichen Ersteinschätzung (entsprechend der IVENA-Nomenklatur: SK1 = Notfall, SK2 = stationäre Versorgung, SK3 = ambulante Versorgung) sowie zur notärztlichen Begleitung. Die korrespondierenden soziodemographischen bzw. klinischen Routinedaten der Fälle wurden aus dem Patientendatenmanagementsystem (SAP i.sh.med) extrahiert, mit den präklinischen Daten manuell zusammengeführt und anonymisiert. Weiterhin wurden die in der ZNA eingesetzten Ressourcen (diagnostische und therapeutische Maßnahmen, die über die körperliche Untersuchung sowie einfache Medikations- und Wundversorgungsmaßnahmen hinausgehen) extrahiert und analog zu ihrer Berücksichtigung im Rahmen der Ersteinschätzung mittels Emergency Severity Index (ESI) ausgewiesen. Die behandelnden Fachrichtungen innerhalb der ZNA wurden fachübergreifend unter „konservativ“ (Innere Medizin, Allgemeinmedizin, Neurologie), „chirurgisch“ (Unfallchirurgie, Viszeralchirurgie, Mund-Kiefer-Gesichtschirurgie, Neurochirurgie, Herz-Thorax-Gefäßchirurgie, plastische Chirurgie/Hand- und Wiederherstellungschirurgie, Augenheilkunde, Urologie, Hals-Nasen-Ohrenheilkunde, Dermatologie) und „psychiatrisch“ (Psychiatrie, Psychosomatik) zusammengefasst.

### Analyse

Die statistische Auswertung erfolgte mit SPSS Statistics 28 (IBM Corporation, Armonk, NY, USA). Deskriptive Ergebnisse wurden anhand von Häufigkeiten, prozentualen Angaben, Mittelwert und Standardabweichung (SD) beschrieben. Gruppenunterschiede wurden mittels Mann-Whitney-U-Test für stetige, nichtnormalverteilte Variablen, χ^2^-Test nach Pearson für kategoriale Variablen bzw. Linear-Trend-Test für ordinalskalierte kategoriale Variablen analysiert. Ein *p*-Wert < 0,05 galt als signifikant. Positive und negative prädiktive Werte wurden unter Verwendung der 4‑Felder-Tafel von OpenEpi, Version 3, ermittelt.

## Ergebnisse

Im 2‑wöchigem Beobachtungszeitraum wurden insgesamt 570 Patienten durch den Rettungsdienst zugeführt (Angaben zum Gesamtkollektiv in Tab. 1).

**Tab. 1:** Gesamtkollektiv rettungsdienstlicher Zuweisungen (*N* = 570)

	Anzahl	%^b^
*Geschlecht (n* *=* *570)*		
Weiblich	283	50,4
Männlich	287	49,6
*Alter* [Jahre] (SD)	60,0 (24,84)	
*Alterskategorien [Jahre] (n* *=* *570)*		
≤ 19	43	7,5
20–29	50	8,8
30–39	52	9,1
40–49	42	7,4
50–59	69	12,1
60–69	67	11,8
70–79	75	13,2
80–89	124	21,8
≥ 90	48	8,4
*Sichtungskategorie (n* *=* *570)*		
SK1	67	11,8
SK2	433	76,0
SK3	70	12,3
*Transport mit notärztlicher Begleitung (n* *=* *570)*		
Nein	525	92,1
Ja	45	7,9
*ESI-Kategorie (n* *=* *521)*^*a*^		
1	12	2,3
2	60	11,5
3	250	48,0
4	187	35,9
5	12	2,3
*Fachbereich innerhalb der ZNA (n* *=* *570)*		
Konservativ	239	41,9
Chirurgisch	303	53,2
Psychiatrisch	28	4,9
*Weiterversorgung (n* *=* *570)*		
Ambulant	307	53,9
Stationäre Aufnahme (intern oder extern)	260	45,6
Verstorben in ZNA	3	0,5

^a^Abweichungen aufgrund fehlender Werte

^b^Rundungsbedingt ergeben die Summen der Prozentangaben nicht immer 100 %

*SD* Standardabweichung, *SK* Sichtungskategorie, *ESI* Emergency Severity Index

Für alle zugeführten Fälle war eine rettungsdienstliche Ersteinschätzung dokumentiert, wobei die Patienten überwiegend der SK2 zugeordnet waren (433/76,0 %). Eine klinische Ersteinschätzung lag für 521 Patienten (91,4 %) vor. Mehrheitlich wurden die Patienten in die mittlere Dringlichkeitskategorie ESI 3 (250/48,0 %) eingestuft, weitere 199 Patienten (38,2 %) entfielen auf die als „niedrig“ definierten Dringlichkeitskategorien ESI 4 und ESI 5. Einer chirurgischen Fachdisziplin wurden 303 Patienten (53,2 %) zugeordnet, durch ein konservatives Fach wurden 239 Patienten (41,9 %) versorgt. Ambulant verblieben 307 Patienten (53,9 %), für 260 (45,6 %) war eine stationäre Aufnahme erforderlich, 3 Patienten verstarben in der ZNA.

### Vergleich von präklinischer Sichtungskategorie, klinischer Ersteinschätzung und Art der Weiterversorgung

**Abb. 1:**
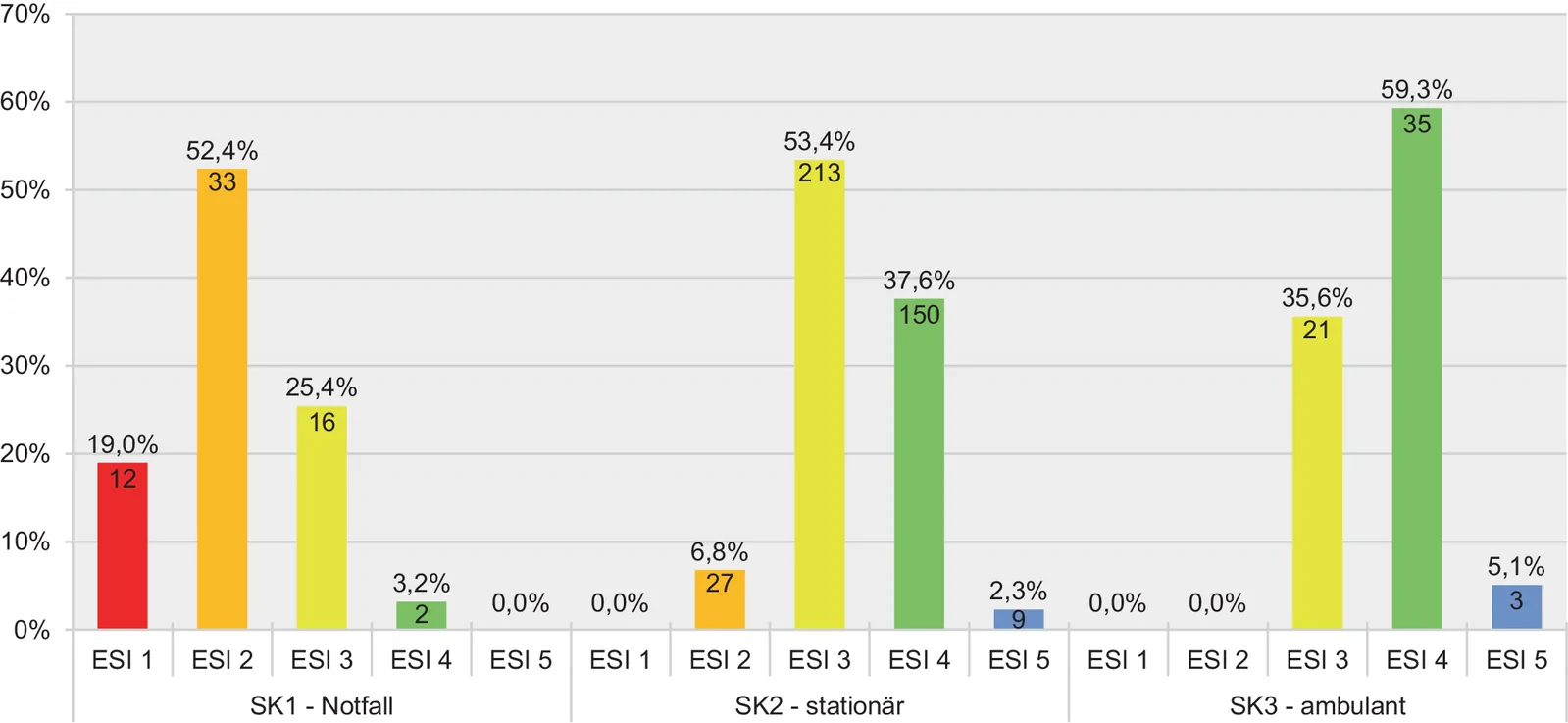
Präklinische Sichtungskategorie und klinische Ersteinschätzung. *SK* Sichtungskategorie, *ESI* Emergency Severity Index

**Abb. 2:**
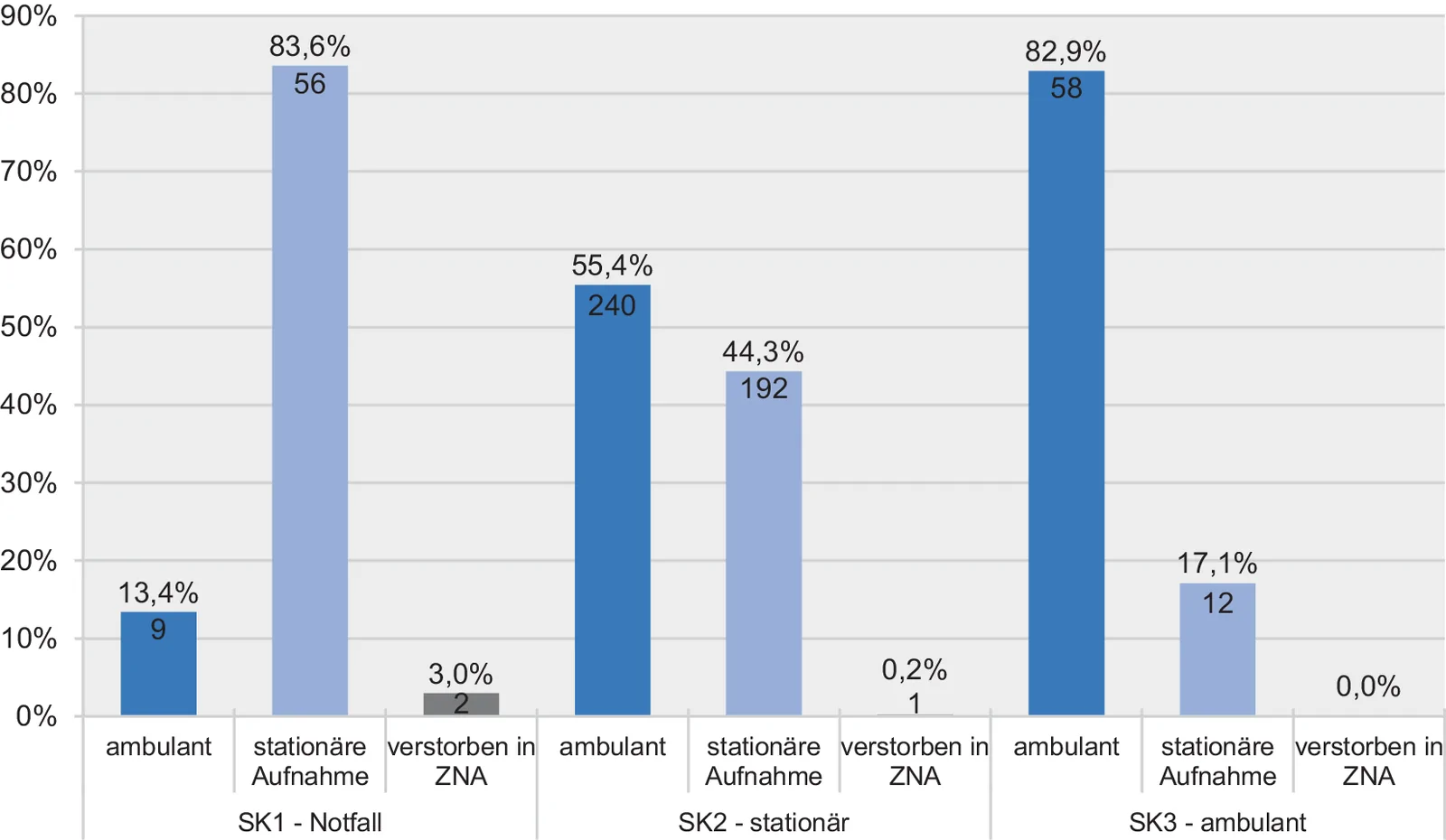
Präklinische Sichtungskategorie und Art der Weiterversorgung. *SK* Sichtungskategorie, *ZNA* zentrale Notaufnahme

Die Dringlichkeit der klinischen Ersteinschätzung korrespondierte mit der präklinischen Sichtungskategorie (Abb. 1): Innerklinisch ESI-1-triagierte Fälle fanden sich ausschließlich in der präklinischen SK1, während als ESI‑5 eingeschätzte Patienten ausschließlich aus der SK2 bzw. SK3 entstammten. Als ESI‑3 und ESI‑4 triagierte Patienten gingen aus allen 3 rettungsdienstlichen Sichtungskategorien hervor. Ein ähnliches Bild zeigt der Vergleich von präklinischer Sichtungskategorie und Art der Weiterversorgung: Je dringlicher die präklinische Ersteinschätzung, desto höher war der Anteil stationärer Aufnahmen (Abb. 2). Von 67 Patienten, die präklinisch als SK1 eingestuft waren, wurden 56 Patienten (83,6 %) stationär aufgenommen, von den 433 Patienten mit SK2 wurden 192 Patienten (44,3 %) stationär aufgenommen. Mit einem Anteil von 82,9 % blieben SK3-Patienten überwiegend ambulant, unter ihnen war kein Todesfall in der Notaufnahme zu verzeichnen. Kumulativ stimmte für 309 von 570 (54,2 %) Patienten die rettungsdienstliche Ersteinschätzung mit der Behandlungsart (stationär/ambulant) nach Versorgung in der Notaufnahme überein. In 249 Fällen (43,7 %) wurde der Behandlungsbedarf bezüglich der Weiterversorgung überschätzt, in 12 Fällen (2,1 %) unterschätzt.

### Vorhersage der Art der Weiterversorgung aus der Sichtungskategorie

Die Berechnung des positiven prädiktiven Werts (PPV) ergab für die SK1 („Notfallversorgung“) und den tatsächlichen Verbleib nach Zuführung in die Notaufnahme eine Wahrscheinlichkeit von 86,6 % für eine stationäre Aufnahme. Für die SK2 („stationäre Versorgung“) zeigte sich ein PPV von 44,6 % für eine stationäre Aufnahme. Bei SK3 („stationäre Aufnahme wahrscheinlich nicht erforderlich“) ergab sich ein negativer prädiktiver Wert von 82,7 % für einen ambulanten Verbleib.

### Charakteristika ambulant und stationär verbleibender Patienten

Sowohl beim Anteil notärztlich begleiteter Transporte, der präklinischen SK, der Ersteinschätzung in der ZNA sowie der Fachbereichszuordnung zeigten sich signifikante Unterschiede zwischen den verglichenen Gruppen (jeweils *p* < 0,001; Tab. 2). Ambulant verbliebene Patienten wurden weniger häufig notärztlich begleitet (9/2,9 % vs. 34/13,1 %) und waren präklinisch überwiegend der SK 2 (240/78,2 %) und SK 3 (58/18,9 %) zugeordnet. Innerklinisch wurden sie mehrheitlich nach ESI 4 bzw. ESI 5 triagiert (153/55,0 %). Stationär aufgenommene Patienten befanden sich am häufigsten in den präklinischen SK 1 (56/21,5 %) und SK 2 (192/73,8 %). Innerklinisch waren sie mehrheitlich in die mittlere Dringlichkeitskategorie ESI 3 eingestuft worden (133/55,4 %). Insgesamt zeigte die innerklinische Ersteinschätzung für Kategorien höherer Behandlungsdringlichkeit korrespondierend einen jeweils höheren Anteil stationärer Aufnahmen. So stiegen die anteiligen Aufnahmeraten von 16,7 % aus ESI 5 über 23,5 % (ESI 4), 53,2 % (ESI 3) und 86,4 % (ESI 2) auf 100 % aus ESI 1 an. Die stationär aufgenommenen Patienten entstammten mehrheitlich aus den konservativen Fachrichtungen (147/56,5 %), während ambulant verbliebene Patienten in der ZNA überwiegend chirurgisch geführt wurden (209/68,1 %).

**Tab. 2:** Patientenkollektiv – Vergleich ambulanter und stationärer Verbleib (*N* = 567)^a^

	Ambulant		Stationär		*p*
	Anzahl	%^b^	Anzahl	%^b^	
*Geschlecht*	*(n* *=* *307)*	–	*(n* *=* *260)*	–	0,640
Weiblich	156	50,8	127	48,8	
Männlich	151	49,2	133	51,2	
*Alter,* Jahre (SD)	58,1 (26,65)		62,2 (22,32)		0,170
*Alterskategorien *(Jahre)	*(n* *=* *307)*	–	*(n* *=* *260)*	–	–
≤ 19	34	11,1	9	3,5	
20–29	28	9,1	21	8,1	
30–39	32	10,4	20	7,7	
40–49	21	6,8	21	8,1	
50–59	31	10,1	37	14,2	
60–69	31	10,1	36	13,8	
70–79	37	12,1	38	14,6	
80–89	62	20,2	62	23,8	
≥ 90	31	10,1	16	6,2	
*Sichtungskategorie*	*(n* *=* *307)*	–	*(n* *=* *260)*	–	< 0,001
SK1	9	2,9	56	21,5	
SK2	240	78,2	192	73,8	
SK3	58	18,9	12	4,6	
*Transport mit notärztlicher Begleitung*	*(n* *=* *307)*	–	*(n* *=* *260)*	–	< 0,001
Nein	298	97,1 %	226	86,9	
Ja	9	2,9 %	34	13,1	
*ESI-Kategorie*	*(n* *=* *278)*^*a*^	–	*(n* *=* *240)*	–	< 0,001
1	–		10	4,2	
2	8	2,9	51	21,3	
3	117	42,1	133	55,4	
4	143	51,4	44	18,3	
5	10	3,6	2	0,8	
*Fachbereich innerhalb der ZNA*	*(n* *=* *307)*	–	*(n* *=* *260)*	–	< 0,001
Konservativ	89	29,0	147	56,5	
Chirurgisch	209	68,1	94	36,2	
Psychiatrisch	9	2,9	19	7,3	

^a^Abweichung von der Gesamtanzahl aufgrund Nichtberücksichtigung der in der ZNA verstorbenen Patienten

^b^Rundungsbedingt ergeben die Summen der Prozentangaben nicht immer 100 %

*SD* Standardabweichung, *SK* Sichtungskategorie, *ESI* Emergency Severity Index

### Ressourcenbedarf ambulant verbliebener Patienten

Für alle ambulant verbliebenen Patienten waren Angaben zu den jeweils eingesetzten Ressourcen verfügbar. Insgesamt waren bei 45 ambulant verbliebenen Patienten (14,7 %) keine weiterführenden diagnostischen oder therapeutischen Maßnahmen in der Notaufnahme erforderlich (Tab. 3). Entsprechend erhielten 262 Patienten (85,3 %) über die Anamnese und körperlichen Untersuchung hinausgehend mindestens eine weitere diagnostische und/oder therapeutische Maßnahme, bevor die Entscheidung zugunsten einer ambulanten Weiterversorgung getroffen wurde. Am häufigsten waren dies Röntgenaufnahmen (118/38,4 %) und Laboruntersuchungen (103/33,6 %). Eine Computer- oder Magnetresonanztomographie wurde in 82 Fällen (26,7 %) angeordnet, für 59 Patienten (19,2 %) wurden durch die versorgende Fachrichtung Konsile weiterer Fachdisziplinen innerhalb der ZNA angefordert. Für Patienten einer konservativen Fachrichtung kamen im Mittel 2,6 Ressourcen zum Einsatz und damit signifikant mehr als für chirurgische Patienten mit durchschnittlich 1,8 Ressourcen (*p* < 0,001). Die gesonderte Betrachtung der präklinisch als SK3 eingestuften und abschließend ambulant verbliebenen Patienten (*n* = 58) zeigte, dass aus dieser Gruppe 12 Patienten (20,7 %) ohne weitere Maßnahmen nach Erstversorgung aus der Notaufnahme entlassen werden konnten (Tab. 4). Bei 28 Patienten (48,2 %) wurden 2 oder mehr Ressourcen vor Entlassung in die ambulante Versorgung eingesetzt. Röntgenaufnahmen erfolgten bei 16 Patienten (27,6 %), 12 Patienten (20,7 %) erhielten Laboruntersuchungen, bei 11 Patienten (19,0 %) wurde eine Computertomographie durchgeführt und für ebenfalls 11 Patienten Konsile mindestens einer weiteren Fachdisziplin angefordert.

**Tab. 3:** Art und Anzahl benötigter Ressourcen ambulant verbliebener Patienten

	Ambulant gesamt (*n* = 307)		Konservativ (*n* = 89)		Chirurgisch (*n* = 209)		Psychiatrisch (*n* = 9)	
	Anzahl	%	Anzahl	%	Anzahl	%	Anzahl	%
*Art der Ressource*								
Konsil	59	19,2	22	24,7	35	16,7	2	22,2
Medikation, subkutan	12	3,9	3	3,4	9	4,3	0	0
Medikation, intramuskulär	14	4,6	0	0	14	6,7	0	0
Medikation, intravenös	29	9,4	16	18,0	13	6,2	0	0
Sonographie	5	1,6	4	4,5	1	0,5	0	0
MRT	11	3,6	7	7,9	4	1,9	0	0
CT	71	23,1	10	11,2	60	28,7	1	11,1
Röntgenuntersuchung	118	38,4	29	32,6	89	42,9	0	0
EKG	68	22,1	59	66,3	7	3,3	2	22,2
Labor	103	33,6	73	82,0	29	13,9	1	11,1
Rezept	55	17,9	14	15,7	41	19,6	0	0
*Anzahl benötigter Ressourcen*								
0	45	14,7	7	7,9	32	15,3	6	66,7
1	75	24,4	10	11,2	64	30,6	1	11,1
2	79	25,7	21	23,6	57	27,3	1	11,1
3	69	22,5	35	39,3	33	15,8	1	11,1
4	28	9,1	11	12,4	17	8,1	0	0
5	7	2,3	3	3,4	4	1,9	0	0
6	3	1,0	1	1,1	2	1,0	0	0
7	1	0,3	1	1,1	0	0	0	0

*MRT* Magnetresonanztomographie, *CT* Computertomographie, *EKG* Elektrokardiographie

**Tab. 4:** Art und Anzahl benötigter Ressourcen ambulant verbleibender Patienten der Sichtungskategorie SK3

	SK 3 & ambulant (*n* = 58)	
	Anzahl	%
*Art der Ressourcen*		
Konsil	11	19,0
Medikation, subkutan	2	3,4
Medikation, intramuskulär	4	6,9
Medikation, intravenös	2	3,4
Sonographie	0	0
MRT	1	1,7
CT	11	19,0
Röntgenuntersuchung	16	27,6
EKG	5	8,6
Labor	12	20,7
Rezept	8	13,8
*Anzahl benötigter Ressourcen*		
0	12	20,7
1	18	31,0
2	13	22,4
3	12	20,7
4	2	3,4
5	1	1,7
6	0	0
7	0	0

*SK* Sichtungskategorie, *MRT* Magnetresonanztomographie, *CT* Computertomographie, *EKG* Elektrokardiogramm

## Diskussion

In der vorliegenden Studie wurde die präklinische Vorhersagbarkeit des Patientenverbleibs (ambulant/stationär) rettungsdienstlich in die Notaufnahme einer Universitätsklinik zugewiesener Patienten sowie der innerklinische Ressourcenverbrauch der abschließend ambulant verbliebenen Rettungsdienstpatienten untersucht. Die rettungsdienstlich prognostizierte und tatsächliche Weiterversorgung stimmte hierbei nur in etwa der Hälfte der Fälle überein, wobei der stationäre Behandlungsbedarf häufig überschätzt wurde.

Mit 54 % wurde mehr als die Hälfte der rettungsdienstlich zugewiesenen Patienten in die ambulante Weiterversorgung entlassen. Dieser Anteil ist kongruent zu Daten aus einer ebenfalls großstädtischen, universitären Notaufnahme, die einen ambulanten Verbleib von 55,2 % der per Rettungsdienst eingelieferten Patienten aufwies [15]. International werden stationäre Aufnahmeraten nach Rettungsdiensttransport von 30–77 % berichtet [6, 8, 16], wobei die Ergebnisse angesichts unterschiedlicher Strukturen der Gesundheitsversorgung kaum vergleichbar sind.

Der Vergleich von ambulanten und stationär aufgenommenen Patienten zeigte für solche mit Aufnahmeindikation eine signifikant häufigere notärztliche Transportbegleitung, eine sowohl präklinisch als auch innerklinisch höhere Dringlichkeitseinschätzung und eine häufigere Zuordnung zu konservativen Fachbereichen innerhalb der Notaufnahme. Die erhöhte Rate notärztlich begleiteter Transporte bei Patienten mit stationärer Aufnahmeindikation deckt sich mit einer Analyse von Krankenhausroutinedaten [5] sowie einer Untersuchung zur Abhängigkeit der stationären Aufnahmerate von der Art der Zuweisung [4]. Eine hinreichende Abgrenzung von ambulanter und stationärer Weiterversorgung gelingt allerdings basierend auf dem Kriterium der notärztlichen Transportbeteiligung nicht. Sowohl unsere Daten als auch weitere Studien [4, 5, 27] weisen notärztlich begleitete Fälle auf, die aus der Notaufnahme in die ambulante Versorgung entlassen werden konnten.

### Präklinische Sichtungskategorie und Art der Weiterbehandlung

Zur Entlastung der Notaufnahmen empfiehlt der Sachverständigenrat im Gutachten „Bedarfsgerechte Steuerung der Gesundheitsversorgung“, dem Rettungsdienst die Möglichkeit einzuräumen, Patienten direkt einer geeigneten ambulanten Versorgungsstruktur zuzuführen, anstatt sie in die Notaufnahme eines Krankenhauses zu transportieren [19].

Der in dieser Studie hohe Anteil ambulant verbliebener Patienten unter den rettungsdienstlichen Zuweisungen in die Notaufnahmen lässt zunächst auf ein hohes Potenzial an Patienten schließen, die bereits primär der ambulanten Versorgung hätten zugeführt werden können. Eine solche zielgerichtete Steuerung bereits durch den Rettungsdienst setzt jedoch eine valide präklinische Einschätzung der Behandlungsdringlichkeit sowie des geeigneten Behandlungsorts voraus, um eine potenzielle Patientengefährdung zu vermeiden. Eine Übereinstimmung zwischen rettungsdienstlicher Einschätzung und tatsächlicher Art der Weiterversorgung nach Behandlung in der Notaufnahme konnte in der vorliegenden Studie jedoch nur in knapp über 50 % der Fälle sowie hierbei insbesondere in den beiden Randgruppen SK1 und SK3 nachgewiesen werden, die die besonders schweren und leichten Fälle beinhalten. In den Fällen ohne Übereinstimmung wurde der Bedarf nach einer stationären Weiterbehandlung zumeist überschätzt. Diese Ergebnisse stehen im Einklang mit einer retrospektiven Studie zur Qualität rettungsdienstlicher Dringlichkeitsbeurteilungen, die zwar eine insgesamt geringere Diskrepanz zwischen präklinischer IVENA-Sichtungskategorie und tatsächlicher Behandlungsart zeigte, bei Abweichungen jedoch zumeist ebenfalls eine Überschätzung des Behandlungsbedarfs berichtete [27]. Die in unserer Studie höhere Diskrepanz könnte durch den größeren Anteil von Patienten in SK2 (76,0 % vs. 51,9 % [27]) bedingt sein, die anfälliger für Fehleinschätzungen zu sein scheint als die beiden Gruppen SK 1 und 3. Als Ursache für die Fehleinschätzungen wird zum einen das Fehlen standardisierter Algorithmen für die Vergabe der Sichtungskategorien im IVENA-System diskutiert [27]. Zum anderen ist die Entscheidungsfindung bezüglich der angemessenen Versorgungsebene, soweit dies bei der großen Vielfalt bundeslandspezifischer Ausbildungscurricula beurteilbar ist, nicht Bestandteil der Ausbildung von Mitarbeitenden im Rettungsdienst [12].

Auch internationale Studien beschreiben nur eine geringe bis moderate Übereinstimmung zwischen Vorhersage des Rettungsdiensts und abschließender Patientendisposition. Die berichteten Vorhersagewerte variieren hierbei erheblich mit einem PPV zwischen 16 und 74 % für eine stationäre Aufnahme und einem NPV zwischen 75 und 90 % für einen ambulanten Verbleib. Diese Befunde deuten auf eine eingeschränkte Fähigkeit des Rettungsdiensts hin, die Notwendigkeit einer stationären Aufnahme bereits präklinisch zuverlässig einzuschätzen [1, 2, 14]. Unsere Ergebnisse zum PPV der SK2 (45 %) und zum NPV der SK3 (83 %) liegen im Bereich der international ermittelten Werte. Vergleichbare Daten zu positiven und negativen Vorhersagewerten fehlen bislang für Deutschland.

Für die präklinische Ersteinschätzung in den Telefonzentralen der kassenärztlichen Vereinigungen sowie für den Einsatz am „gemeinsamen Tresen“ von Bereitschaftsdienstpraxen und Notaufnahmen wurde die Software „Strukturierte medizinische Ersteinschätzung in Deutschland (SmED)“ entwickelt [28] und im Rahmen des vom Innovationsfonds geförderten Projekts DEMAND evaluiert. Erste Ergebnisse zur Reduktion von ambulanten Notaufnahmekontakten zeigten sich dabei vielversprechend [7], kürzlich publizierte Studien zur Machbarkeit sowie zur Patientensicherheit in der Zusammenschau dagegen ambivalent. Während der Einsatz von SmED am gemeinsamen Tresen als machbar bewertet wurde [11, 17], wird insbesondere eine nicht zeitnahe Weiterleitung von Patienten in vertragsärztliche Versorgungsstrukturen unter dem Gesichtspunkt der Patientensicherheit insgesamt deutlich kritischer bewertet [25]. Bisherige Studien zum Einsatz von SmED zielten dabei jeweils auf selbsteinweisende Patienten, die fußläufig die Notaufnahme erreichten, ab. Der Einsatz von SmED zur Unterstützung der rettungsdienstlichen Entscheidung hinsichtlich Versorgungszeitpunkt und -ort wird derzeit innerhalb von regionalen Pilotprojekten geplant bzw. erprobt [29]. Die Ergebnisse hierzu stehen noch aus.

### Ressourcenbedarf

Mehr als die Hälfte der ambulant verbliebenen Patienten erhielt im Rahmen der Versorgung in der Notaufnahme mindestens 2 diagnostische und/oder therapeutische Ressourcen. Für Deutschland existieren bislang kaum vergleichbare Daten zum diagnostischen Bedarf dieser Patientengruppe. Eine Studie zu ärztlich eingewiesenen, aber letztlich ambulant verbliebenden Notaufnahmepatienten zeigte, dass 70 % eine Blutentnahme, 18 % eine Röntgenuntersuchung und 9 % eine Computertomographie erhielten, bevor die Entscheidung für eine ambulante Weiterversorgung getroffen werden konnte [18].

Für knapp 20 % der ambulant verbliebenen Patienten in der vorliegenden Studie waren konsiliarische Leistungen dokumentiert, sodass vor Entlassung mindestens eine weitere Fachdisziplin eingebunden wurde. Möglicherweise spiegelt sich hierin die zunehmende Multimorbidität älterer Notfallpatienten mit komplexer werdenden Beschwerdebildern wie sie national und international beschrieben werden, wider [13, 26]. Eine direkte rettungsdienstliche Zuweisung dieser Patienten in ambulante Strukturen wäre vielfach wenig praktikabel, da sie vermutlich oft den Besuch mehrerer Facharztpraxen erfordern würde und eine zeitnahe Ausschlussdiagnostik kaum möglich wäre.

Nur etwa 15 % aller ambulant verbliebenen Patienten unserer Studie erhielten keine weiterführenden Ressourcen. Betrachtet man ausschließlich die initial als SK3 eingestuften und ambulant verbliebenen Patienten, reduziert sich dieser Anteil auf rund 2 %, die sicher und sinnvoll direkt in eine hausärztliche Praxis hätten disponiert werden können – allerdings ohne Berücksichtigung der Praxisöffnungszeiten.

### Schlussfolgerung

Unsere Ergebnisse stehen im Einklang mit früheren Studien, die das Potenzial einer primären Umsteuerung niedrigdringlicher Rettungsdienstpatienten in ambulante Versorgungsstrukturen als begrenzt hinsichtlich einer spürbaren Entlastung von Notaufnahmen einschätzen [9, 15]. Neben der aufgezeigten Unsicherheit in der präklinischen Einschätzung des weiteren Versorgungsbedarfs stützten unsere Ergebnisse die Bedenken, dass die für eine adäquate Versorgung erforderlichen diagnostischen und therapeutischen Ressourcen derzeit möglicherweise selbst während regulärer Sprechzeiten in der ambulanten vertragsärztlichen Versorgung nicht flächendeckend, unmittelbar und regelhaft verfügbar sind. Inwieweit künftig neue strukturierte Ersteinschätzungsverfahren die Umsetzung sektorübergreifender Zuweisungen unterstützen können, ist bislang nicht abschließend geklärt und Gegenstand aktueller Untersuchungen.

### Limitationen

Bei der Einordnung der Ergebnisse sowie deren Übertragbarkeit sind einige Limitationen zu berücksichtigen. Die Zusammensetzung des Patientenkollektivs einer Notaufnahme ist abhängig von den örtlich gegebenen Versorgungsstrukturen und der aktuellen Auslastungssituation der regional verfügbaren Krankenhausnotaufnahmen mit den daraus resultierenden Optionen, eine spezifische Notaufnahme rettungsdienstlich anzusteuern. In diesem Zusammenhang kann auch der Status als Universitätsklinikum und Supramaximalversorger zu einer Selektion des Patientenkollektivs beitragen und somit die Vergleichbarkeit beeinflussen. Mit Bezug auf die eingesetzten Ressourcen ist zu berücksichtigen, dass diese zwar aus Sicht des notfallmedizinischen Personals (Notfallpflege oder ärztliches Personal) zum Zeitpunkt der Ersteinschätzung und Versorgung in der Notaufnahme erforderlich erschienen, retrospektiv jedoch nicht eindeutig belegbar ist, ob diese Maßnahmen tatsächlich zwingend notwendig waren, um über die weiterführende Versorgungsebene entscheiden zu können. Aufgrund der monozentrischen Konzeption der Studie in Verbindung mit einer relativ kurzen Beobachtungszeit ist die Übertragbarkeit der Ergebnisse somit möglicherweise eingeschränkt. Aufgrund der fehlenden Möglichkeit zur formalen Überprüfung der Validität der präklinischen SK-Klassifikation ist weiterhin zu ergänzen, dass die Eignung dieses Systems für eine sektorübergreifende Steuerung in der vorliegenden Studie, trotz der beobachteten Korrespondenz mit der klinischen Ersteinschätzung sowie der stationären Aufnahmerate, nicht abschließend beurteilt werden kann.

## Fazit für die Praxis

- Rettungsdienstliche Sichtungskategorien alleine erlauben keine sichere Aussage über die Art der Weiterversorgung nach Behandlung in der Notaufnahme.
- Auch ambulant verbliebende Notaufnahmepatienten benötigen mitunter umfassende und komplexe diagnostische und therapeutische Ressourcen und/oder ein interdisziplinäres Behandlungsteam.
- Auf Basis aktueller Daten ist eine direkte rettungsdienstliche Zuführung von Patienten in den ambulanten Sektor unter Patientensicherheitsaspekten kritisch zu betrachten. Eine signifikante Entlastung von Notaufnahmen ist hierdurch nicht zu erwarten.

## Data Availability

Die erhobenen Datensätze können auf begründete Anfrage in anonymisierter Form beim korrespondierenden Autor angefordert werden Die Daten befinden sich auf einem Datenspeicher der Medizinischen Hochschule Hannover
